# Supplementary material for: Bidirectional Mendelian Randomization Analysis of the Causal Relationship Between Uterine Fibroids and Breast Cancer in East Asian Women
Source: Biomedicines. 2025 Oct 29;13(11):2654. doi: 10.3390/biomedicines13112654 (PMC12650747; doi:10.3390/biomedicines13112654)
Supplement: Supplementary file 1 [file biomedicines-13-02654-s001.zip › Additional File S1.pdf]

Bidirectional Mendelian Randomization Analysis of the Causal Relationship Between Uterine Fibroids and Breast Cancer in East Asian Women

A supplementary two-sample Mendelian randomization (MR) analysis was conducted using the BioBank Japan (BBJ) uterine fibroid (UF) GWAS as the exposure and the Korean Genome and Epidemiology Study (KoGES) breast cancer (BC) GWAS as the outcome (**Supplementary Table S3**). 16 independent SNPs were retained as instrumental variables, with a mean F-statistic of 79.20, indicating that weak instrument bias was unlikely.

Table S3. Summary statistics of data source.

Traits	Data source	No. of participants	Population	No. of Variants	URL
Uterine fibroids	BBJ	80,208 (14,475 cases + 65,733 controls)	East Asian	13,401,454	https://pheweb.jp/
Breast cancer	KoGES	46,330 (424 cases + 45,906 controls)	East Asian	7,982,452	https://koges.leelabsg.org/

Abbreviations: BBJ, BioBank Japan; KoGES, Korean Genome and Epidemiology Study.

Instrument validity is summarized in **Supplementary Table S4**. There was no evidence of heterogeneity (Cochran's Q, $p = 0.535$; Rücker's Q′, $p = 0.472$). The MR-PRESSO global test did not detect outliers ($p = 0.527$). The MR-Egger intercept was not significant ($\beta = -0.019$, $p = 0.682$), and the SIMEX-adjusted intercept was similarly non-significant ($\beta = -0.021$, $p = 0.664$), suggesting that unbalanced horizontal pleiotropy was unlikely to bias the results.

Table S4. Heterogeneity and horizontal pleiotropy of instrumental variables.

Exposure	Outcome				Heterogeneity		Horizontal pleiotropy				
					Cochran's Q test from IVW	Rücker's Q' test from MR-Egger	MR-PRESSO global test	MR-Egger		MR-Egger (SIMEX)	
		N	*F*	I^2 (%)	*p*	*p*	*p*	Intercept, *β* (SE)	*p*	Intercept, *β* (SE)	*p*
Uterine fibroid	Breast cancer	16	79.20	87.33	0.535	0.472	0.527	−0.019 (0.046)	0.682	−0.021 (0.048)	0.664

Abbreviations: β, beta coefficient; *F*, mean *F*-statistic; IVW, inverse-variance-weighted; MR, Mendelian randomization; N, number of instruments; PRESSO, pleiotropy sum of residuals and outlier; SE, standard error; SIMEX, simulation extrapolation.

Causal estimates are presented in **Supplementary Figure S1**. Across all MR methods, genetically predicted UF showed no significant association with BC. The IVW estimate suggested a weak negative effect (OR = 0.937; 95% CI: 0.701–1.251; $p = 0.657$), the weighted median estimate was close to null (OR = 0.991; 95% CI: 0.631–1.556; $p = 0.968$), and MR-Egger and MR-Egger (SIMEX) yielded positive but imprecise estimates with wide confidence intervals (MR-Egger OR = 1.074; 95% CI: 0.529–2.179; $p = 0.846$; SIMEX OR = 1.094; 95% CI: 0.517–2.315; $p = 0.818$). **Supplementary Figure S2** shows the scatter plot of SNP–exposure and SNP–outcome associations. Taken together, these results indicate that genetically predicted UF was not significantly associated with BC risk, and effect directions were inconsistent across MR methods. Replication in larger datasets will be necessary to clarify these findings.

Figure S1. Forest plot of MR estimates for uterine fibroids on breast cancer.

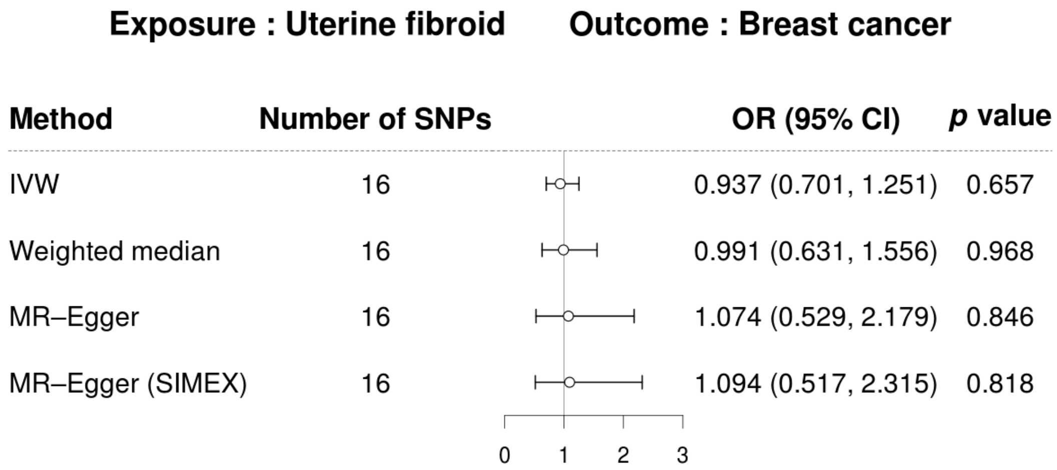

Abbreviations: CI, confidence interval; IVW, inverse-variance weighted; MR, Mendelian randomization; OR, odds ratio; SIMEX, simulation extrapolation; SNP, single-nucleotide polymorphism.

Figure S2. Scatter plot of SNP–uterine fibroids and SNP–breast cancer.

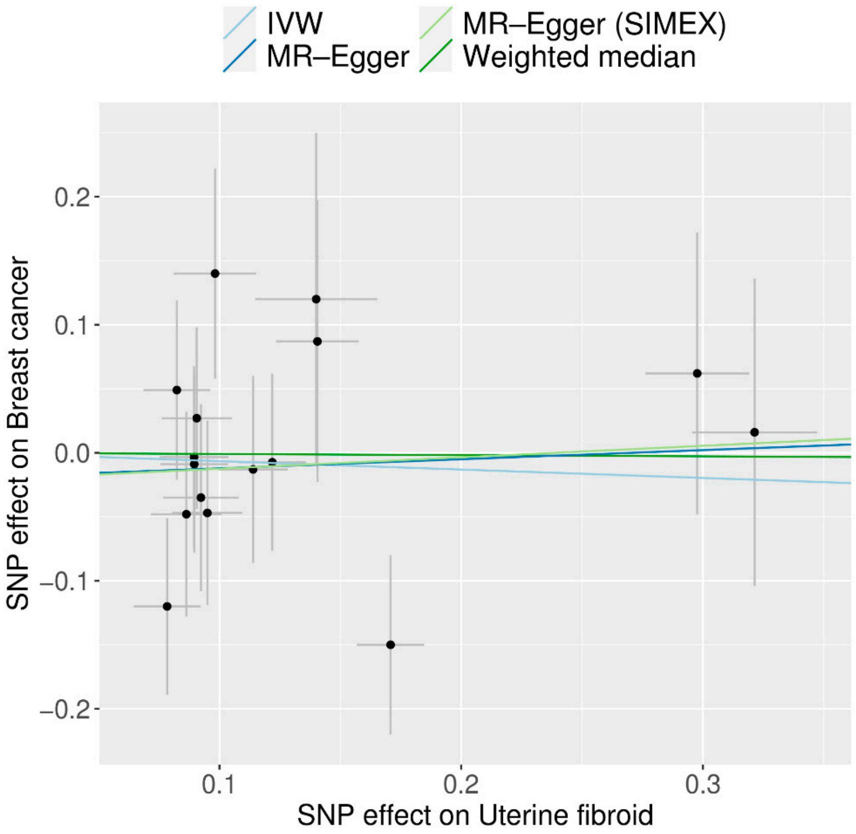

Light blue, dark blue, light green, and dark green regression lines represent the IVW, MR-Egger, MR-Egger (SIMEX), and weighted median estimates, respectively. The slope of the line represents the causal effect of each method. Abbreviations: IVW, inverse-variance-weighted; MR, Mendelian randomization; SIMEX, simulation extrapolation; SNP, single-nucleotide polymorphism.
